# The Impact of Climate Change on Pregnant Women’s Health: A Scoping Review of Reviews

**DOI:** 10.3390/nursrep16090332

**Published:** 2026-09-09

**Authors:** Cristina Álvarez-García, Cesar Ivan Aviles-Gonzalez, Sebastián Sanz-Martos, Carmen Álvarez-Nieto, Isabel M. López-Medina, Sergio Martínez-Vázquez, Maria Dolores López-Medina, Eva M. Montoro-Ramirez

**Affiliations:** 1 Department of Nursing, University of Jaen, 23071 Jaen, Spain; cagarcia@ujaen.es (C.Á.-G.); calvarez@ujaen.es (C.Á.-N.); imlopez@ujaen.es (I.M.L.-M.); emmontor@ujaen.es (E.M.M.-R.); 2 Department of Medicine and Surgery, University of Enna “Kore”, 94100 Enna, Italy; cesar.aviles@unikore.it; 3 Consortium for Biomedical Research in the Epidemiology and Public Health (CIBERESP), 28029 Madrid, Spain; 4 Maternity Unit, University Hospital of Jaén, 23007 Jaen, Spain; mlmedina@ujaen.es

**Keywords:** climate change, environmental health, maternal health, nursing, pregnant women

## Abstract

**Background/Objectives**: Records through early 2026 mark 2025 as the warmest year in over 170 years. Pregnant women are among the populations most vulnerable to climate change. The objective of this study was to conduct a review that synthesizes existing knowledge on how the different dimensions of climate change are affecting the vulnerable population of pregnant women. **Methods**: A scoping review of reviews (PROSPERO: CRD420261400815) was conducted in the Health Sciences databases: PubMed, SCOPUS, PsycInfo, CINAHL and WOS, with no time or language restriction until February 2026. A qualitative synthesis was carried out on data extracted from the different reviews, and a frequency mapping of reported maternal and perinatal outcomes was performed. The methodological quality of the individual reviews included in this review was evaluated using the CASPe tool. **Results**: Air pollution primarily links to developmental issues and gestational hypertension (four reviews), while extreme heat was most frequently reported in relation to preterm birth (eight reviews). Natural disasters mostly compromise maternal mental health (five reviews), vector-borne diseases were most frequently associated with perinatal complications like low birth weight and preterm delivery (three reviews), and chemical exposure is chiefly associated with gestational diabetes (two reviews). **Conclusions**: Given all the maternal and perinatal outcomes reported in relation to climate change, healthcare professionals play a key role in advising pregnant women on their actual and perceived knowledge, risk perception, affective evaluation, self-efficacy, intention to adopt protective behaviors, and subsequent information-seeking behavior.

## 1. Introduction

Global temperature records up to early 2026 indicate that 2025 was the warmest calendar year on record in more than 170 years, marked by escalating climate emergencies ranging from prolonged heatwaves to severe wildfires, floods, droughts, and tropical cyclones. As emphasized by the Intergovernmental Panel on Climate Change (IPCC), the health threats posed by climate change are intrinsically linked to social and economic determinants. These impacts are not distributed evenly; rather, they disproportionately affect vulnerable groups, thereby widening health inequalities—unfair and avoidable differences in health status, life expectancy, and healthcare access [1]. Pregnant women represent a uniquely susceptible population, facing profound health complications from climate-related hazards. However, their specific health needs remain underrepresented in national and global climate adaptation responses [2].

While climate change represents a major dimension of environmental health, this review focuses explicitly on climate change—encompassing extreme heat, climate-driven air pollution, severe weather events, and climate-related toxicant exposures—and its direct and indirect effects on health outcomes during pregnancy. To understand these multidimensional impacts, pregnancy-related exposures must be viewed through an integrated conceptual framework linking climate dimensions through physiological and psychosocial pathways to maternal, obstetric, fetal, neonatal, psychological, and social outcomes.

The heightened vulnerability of pregnant women is rooted in critical physiological adaptations that alter thermoregulation, cardiovascular function, and metabolic demand. Pregnancy significantly increases basal metabolic rate and internal heat production. When exposed to elevated ambient temperatures or extreme heatwaves, a pregnant woman’s thermoregulatory threshold is lowered, rapidly straining sweat production and cutaneous vasodilation. This can result in severe hyperthermia, dehydration, uterine hypoperfusion, and uterine contractions [3]. Concurrently, physiological adaptations—including a 20% increase in oxygen consumption, a 40% to 50% increase in minute ventilation, and a 40% increase in cardiac output—substantially elevate the internal dose and systemic circulation of inhaled environmental pollutants [4]. Climate-exacerbated air pollution, including fine particulate matter (PM_2.5_ and PM_10_), ground-level ozone (O_3_), nitrogen dioxide (NO_2_), carbon monoxide (CO), and hazardous wildfire smoke, directly impairs physiological well-being. These exposures trigger systemic inflammatory cascades, placental vascular constriction, endothelial dysfunction, and oxidative stress, which collectively compromise fetal-placental nutrient exchange.

In addition to thermal and atmospheric hazards, climate change alters patterns of chemical exposure through distinct environmental pathways. Shifting agricultural weather patterns and rising temperatures drive an increased reliance on pesticides, heightening environmental exposure risks for pregnant women in rural and agricultural communities. Severe flooding and sea-level rise mobilize heavy metals, industrial toxicants, and persistent organic pollutants into municipal water systems and agricultural soils. Furthermore, climate-driven wildfires release complex toxic chemical mixtures—including polycyclic aromatic hydrocarbons (PAHs) and heavy metals—that cross the placental barrier, threatening endocrine signalling and organogenesis.

Beyond direct physical and chemical hazards, climate change operates through complex indirect and structural pathways. Extreme weather events, flooding, and severe droughts disrupt food systems and compromise water security, predisposing pregnant women to acute malnutrition, food insecurity, and waterborne pathogens [5]. Shifting climatic conditions also expand the geographic range of vector-borne pathogens (such as Zika virus, malaria, dengue, and Chikungunya), which pose severe risks of maternal mortality, fetal growth restriction, and severe congenital malformations [5].

Simultaneously, exposure to climate disasters places a heavy, lasting burden on maternal psychological well-being. Pregnant women experiencing climate-driven displacements, extreme weather events, or loss of infrastructure face heightened rates of post-traumatic stress disorder (PTSD), generalized anxiety, major depression, and eco-anxiety—a chronic fear of environmental doom. Psychological trauma and extreme stress hyperactivate the maternal hypothalamic–pituitary–adrenal (HPA) axis, elevating cortisol levels and increasing the risk of adverse mood outcomes, neurodevelopmental alterations, and adverse birth outcomes. These mental health burdens and physiological risks do not occur in a vacuum; they intersect heavily with social determinants of health. Black, Indigenous, minoritized, and socioeconomically disadvantaged women experience disproportionate climate exposures due to structural inequities, including living in urban heat islands, reduced access to air conditioning, lower baseline healthcare access, and chronic environmental injustice [6,7,8].

Although a growing body of secondary literature has examined the relationship between climate change and maternal and perinatal health, previous syntheses differ substantially in scope, populations, exposures, and methodological approach. Several reviews have focused on specific climate-related exposures or selected health outcomes, while broader syntheses have also emerged. For example, Conway et al. [9] provided an overview of systematic reviews of climate hazards, air pollution, and maternal and newborn health published through February 2023; however, reviews without a systematic methodology and those focused exclusively on chemical contaminants were excluded, and no eligible reviews addressing climate-sensitive infectious diseases were identified. Other broad narrative work has considered climate change from a wider maternal and child health perspective [10]. The present review is therefore intended to complement these previous syntheses. Specifically, it provides an updated, pregnancy-centred mapping of the secondary evidence through February 2026 across five climate-related exposure dimensions—air pollution, extreme heat, natural disasters, vector-borne diseases, and chemical exposures—and organizes the reported consequences across maternal, obstetric, and fetal/neonatal health domains. In addition, it integrates methodological appraisal, review-level frequency mapping, and a cross-dimensional exposure–outcome matrix to identify areas where secondary evidence is concentrated and where relevant gaps persist. Accordingly, this scoping review of reviews aims to (1) map the scope and nature of climate-related exposures affecting pregnant women; (2) synthesize reported evidence across maternal, obstetric, and fetal/neonatal health domains; and (3) identify critical evidence gaps relevant to future research, prenatal care, and climate adaptation strategies.

## 2. Materials and Methods

### 2.1. Design and Protocol Registration

A scoping review of reviews was conducted in accordance with the methodological guidelines of the Joanna Briggs Institute (JBI) [11]. The review was also conducted in accordance with the PRISMA-ScR guidelines (Supplementary Material File S1: [12]. This review was registered with PROSPERO (CRD420261400815) as an umbrella review. Following initial database searching, the protocol was deviated from by expanding the design to a scoping review. This change was implemented to encompass all review types (narrative, integrative, scoping, systematic, meta-analyses, umbrella) and provide a broader overview of the available literature on this topic. A scoping review of reviews was selected over an umbrella review or systematic review of reviews primarily because our objective was to map the broad extent, range, and nature of existing literature on the impact of climate change on pregnant women’s health, clarify key concepts, and identify overarching evidence gaps. While umbrella reviews generally focus on synthesizing high-quality evidence from systematic reviews to answer narrow, intervention-focused questions, a scoping design allows for a comprehensive mapping of a heterogeneous body of secondary research.

### 2.2. Eligibility Criteria

Inclusion criteria for considering studies for this review were (a) studies discussing the effects of climate change on pregnant women; (b) reviews of any kind (narrative, integrative, scoping, systematic, meta-analyses, umbrella); (c) no restriction on the publication date; and (d) no language restrictions. Exclusion criteria included (a) review protocols; (b) studies that assess only effects on the fetus or on child development; and (c) policy documents or guidelines.

### 2.3. Literature Search and Databases

Two authors of this review (CAG, EMMR) conducted the literature search in the Health Sciences databases: PubMed, SCOPUS, PsycInfo, CINAHL and Web of Science (WOS), until 24 February 2026. In addition, backward citation searching was performed in the reference list of potential studies to be included in the review. Grey literature was also reviewed, such as reports from organisations specialising in maternal and perinatal health, including the International Midwives Confederation Statement, The Conversation and Barcelona Institute of Global Health, as well as the proceedings of various relevant national and international congresses, seminars and conferences relating to the search topic. The search strategy answered the research question: How does climate change affect pregnant women’s health? It was based on the PICOS system (Population: pregnant women; Intervention or Exposure: climate change, including air pollution, extreme heat, natural disasters, vector-borne diseases and chemicals; Comparison: no exposure; Outcome Variables: effects on maternal and perinatal health; Study Design: review) and keywords selected from terms indexed in the PubMed thesaurus (MeSH [Medical Subject Headings]). Table 1 shows the search strategies and the filters used in each database.

### 2.4. Selection Process, Data Extraction and Synthesis

Two reviewers (CAG, EMMR) searched information sources independently and assessed the identified studies for inclusion. Records were managed through the bibliographic manager EndNote (Endnote Web, Clarivate, Philadelphia, PA, USA). The full text of a study was reviewed when it could not be clearly excluded on the basis of its title and abstract, following discussion between the two reviewers. A study was included when both reviewers independently assessed it as satisfying the inclusion criteria from the full text. In case of disagreement, a third reviewer examined the documents following the decision rule identified in the data extraction protocol (SMV).

Data were extracted by two researchers independently (CAG, EMMR). The extracted data were compiled in a data collection sheet, which was developed by consensus among the researchers in Excel (Office 2019, Microsoft Corporation, Redmond, WA, USA). These data corresponded to (1) general characteristics of the study (authors, year of publication, country of origin, review design, number of studies included); (2) exposure type; (3) maternal outcomes; (4) obstetric outcomes; and (5) fetal/neonatal outcomes.

Finally, outcomes and findings were synthesized using a hybrid inductive-deductive thematic approach. Primary deductive outcome domains were broadly categorized a priori using established maternal health frameworks into maternal outcomes, obstetric outcomes and fetal/neonatal outcomes. Inductive sub-themes were coded iteratively as they arose during data extraction. Disagreements regarding data extraction, variable interpretation, or thematic classification were resolved through direct discussion between the two extractors (CAG, EMMR). If consensus was not reached, a third senior author (SMV) served as an arbiter. The final matrix of thematic categories and outcome groupings was reviewed, discussed, and formally ratified by all members of the research team to ensure conceptual coherence. Also, a frequency mapping of reported outcomes of maternal and perinatal outcomes was carried out. We purposefully avoided statistical meta-pooling or quantitative synthesis due to heterogeneous outcome measures and reporting formats across reviews.

Because the eligibility criteria intentionally encompassed heterogeneous review methodologies, review type and methodological quality were explicitly considered during interpretation rather than treating all included reviews as evidentially equivalent. Systematic reviews and meta-analyses were used as the principal sources for quantitative associations and effect estimates when available. Umbrella reviews were considered as tertiary syntheses and interpreted with particular attention to the possibility of overlap among underlying primary studies. Scoping and integrative reviews contributed primarily to mapping the breadth of exposures, outcomes, concepts, and research gaps, whereas narrative, expert, contemporary, and general overview reviews were used mainly to provide contextual and conceptual information. Findings from these latter review types were not used to strengthen the inferred magnitude or certainty of an association. Accordingly, the frequency mapping represents an unweighted descriptive count of reviews reporting a given outcome and should not be interpreted as an evidence grade, pooled effect estimate, measure of certainty, or count of independent primary studies.

Due to heterogeneity in reporting styles across included reviews and the fact that some narrative reviews did not explicitly list the studies included in the review, primary study overlap was not quantitatively mapped via Corrected Covered Area (CCA). Thus, the overlap was addressed qualitatively rather than quantitatively. Data extraction focused on broader theoretical concepts, review-level conclusions, and thematic frameworks rather than pooling numerical data from individual primary studies. Consequently, outcome frequencies represent review-level reporting counts and should not be interpreted as independent replications or additive primary evidence.

### 2.5. Methodological Quality Appraisal

The methodological quality of the individual reviews included in this scoping review of reviews was evaluated using the CASPe tool [13]. The Critical Appraisal Skills Programme (CASP) framework was applied as a structured, common approach to assessing the general aspects of the included reviews. The assessment focused on methodological transparency, clarity of objectives, identification and consideration of the available evidence, acknowledgement of limitations, and the appropriateness of the conclusions. The CASP assessment was intended to support the critical interpretation of the evidence and was not used to establish direct equivalences or specific estimates of the risk of bias between different types of reviews based on their design. This tool has a specific checklist for reviews that assesses three questions: are those results valid?, what are the results?, and are they applicable in your context? The checklist comprises 10 items to be answered with ‘yes’ or ‘no’.

The quality of the reviews was classified following the creators of the tool’s guidelines. The quality was classified as low if the first two assumptions—that the topic was clearly defined and that appropriate articles were included—were not met. The quality of the review was rated as moderate if assumption 4 (assessment of the quality of the included studies) was not met. The quality of the review was rated as moderate-high if assumption 4 was met, but assumption 7 (the accuracy of the results was not determined) was not met. If all the assumptions were met, the quality of the review was determined to be high.

This analysis was performed independently by two researchers (CAG, EMMR), and disagreements were resolved by a third researcher (SMV), as done in every step previously.

## 3. Results

### 3.1. Study Selection

**Figure 1:**
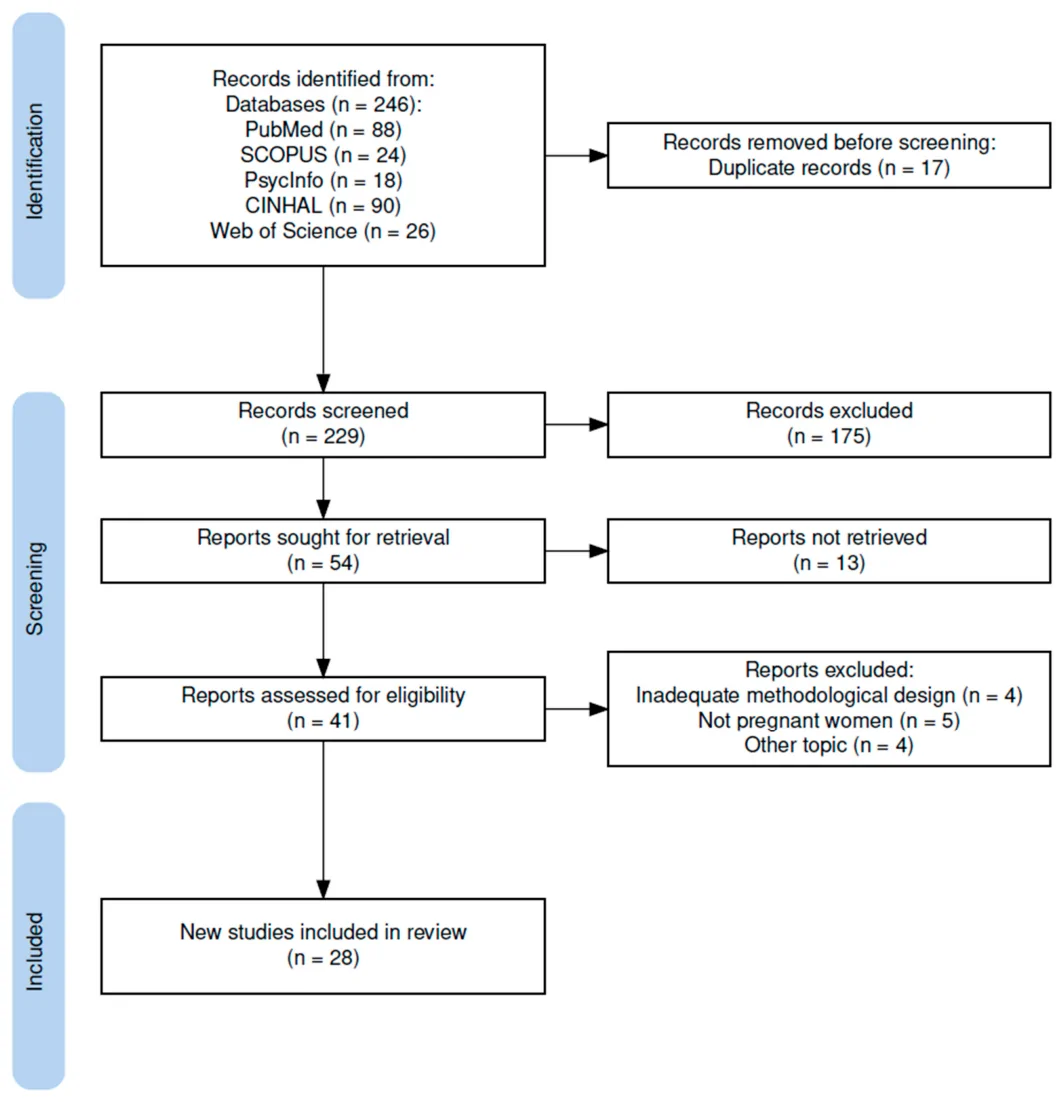
PRISMA flow chart.

A total of 246 studies were retrieved from 5 databases. A literature search for grey literature in the several sources consulted did not identify any review report that could be considered for inclusion in this study. After eliminating 17 studies as duplicates, 229 studies were screened by title/abstract. After this screening, 175 studies were eliminated for not being relevant. Thus, the reports sought for retrieval were 54; nevertheless, of the initial records screened at full text, 13 reports could not be retrieved despite exhaustive efforts. These efforts included utilizing institutional interlibrary loan services, searching public and academic digital archives, and contacting corresponding authors via email and academic networks. The specific reasons for non-retrieval were conference/seminar abstracts without published full-text manuscripts (*n* = 10) and restricted access or broken grey literature repository links (*n* = 3). So, 41 studies were assessed in full text for eligibility. Finally, 28 reviews were included [9,10,14,15,16,17,18,19,20,21,22,23,24,25,26,27,28,29,30,31,32,33,34,35,36,37,38,39] after excluding 4 studies due to their design, 5 due to the population and 4 due to the topic. Figure 1 shows the study selection process.

### 3.2. Characteristics of the Included Studies

Table 2 shows the characteristics of the studies included in the present review. Twenty-eight reviews were included that were carried out between the years 2010 and 2025 in different countries around the world, highlighting 16 reviews in the United States of America [10,16,17,18,19,22,25,26,27,28,30,32,33,35,36,37]. The included reviews provided data from a mean of 40 studies. The areas with the most evidence—due to the higher number of systematic reviews—are natural disasters (with 6 systematic reviews [9,14,15,18,24,27], 2 of which included a meta-analysis [14,24]), extreme heat (with 5 systematic reviews [9,18,19,30,31], 1 of which included a meta-analysis [31]) and air pollution (with 5 systematic reviews [9,18,20,26,33]).

### 3.3. Methodological Quality Appraisal

The methodological quality in each review is detailed in Table 3. Of the 28 studies included in the review, 3 were of high quality [14,24,31], 9 were of moderate-high quality [14,17,18,19,20,26,27,30,33], 16 were of moderate quality [10,16,17,21,22,23,25,28,29,32,34,35,36,37,38,39], and none were of low quality. The criteria that were least frequently met were the failure to assess the quality of the studies included in the review (16 studies) [10,16,17,21,22,23,25,28,29,32,34,35,36,37,38,39] and the failure to determine the precision of the results, as meta-analyses were not carried out for 25 studies [9,10,15,16,17,18,19,20,21,22,23,25,26,27,28,29,30,32,33,34,35,36,37,38,39].

### 3.4. Qualitative Synthesis

Consistent with the synthesis approach described above, quantitative effect estimates are reported only when available from structured evidence syntheses, whereas non-systematic reviews contribute primarily to thematic and contextual mapping rather than to inferences regarding effect magnitude or certainty.

The results were grouped according to the dimensions of climate change that affect pregnant women and are shown below.

#### 3.4.1. Air Pollution

Ten reviews evaluated the impact of air pollution on maternal and perinatal health [9,10,18,20,21,26,29,33,34,36]. Overall, exposure to airborne pollutants—specifically fine particulate matter PM_2.5_, NO_2_, SO_2_, and CO—was consistently linked to severe pregnancy complications and adverse fetal outcomes. Exposure to PM_2.5_ was associated with a 34% higher risk of full-term low birth weight <10th percentile or <2500 g [26] and elevated odds of hypertensive disorders during pregnancy [9,18,21,33] by 47% [9]. Pollutants were also consistently correlated with gestational diabetes [18,21,33], preterm birth, fetal death, abortion, and maternal postpartum depression [29,33,36]. Furthermore, broader exposure to airborne fine particles, heavy metals, and endocrine disruptors was linked to potential deficits in physical, cognitive, and behavioral development in mothers and offspring [10,20,21,34].

#### 3.4.2. Extreme Heat

Twelve reviews examined the impact of extreme temperatures on pregnant women’s health [9,10,16,18,19,23,29,30,31,32,36,38]. Amekpor et al. [10] reported reduced food security associated with heatwaves, while Marudo et al. [32] identified acute heat-related illnesses, including dehydration, heat exhaustion, and heatstroke. Specific maternal conditions reported included an increased likelihood of congestive heart failure [36], maternal mental health issues [18], and placental abruption [16]. At latitudes above 40° N and 40° S, extreme temperatures were linked to lower observed vitamin D synthesis [38].

Regarding pregnancy complications, multiple studies demonstrated increased odds of miscarriages, cardiovascular events [16,18], hypertensive disorders during pregnancy [9,16,18,32] (RR = 1.25; 95% CI = 1.10–1.42) [9], and gestational diabetes [16,18,31,36], (OR = 1.28; 95% CI = 1.05–1.74) [31]. For neonatal and fetal health, studies reported low birth weight and neonatal stress [30,36], a 48% higher odds of congenital anomalies (OR = 1.48; 95% CI = 1.16–1.88) [31], a higher likelihood in fetal death [16,30,31,32,36] by 13% (OR = 1.13; 95% CI = 0.95–1.34) [31], and an increased risk estimate for preterm birth before 37 weeks [16,19,23,29,30,31,32,36] by 4% (OR = 1.04; 95% CI = 1.03–1.06) [31].

#### 3.4.3. Natural Disasters

Eleven reviews investigated the impact of various natural disasters on pregnant women’s health [9,10,14,15,17,18,23,24,27,28,36]. Disasters such as hurricanes and earthquakes were associated with disruptions in basic needs, coinciding with decreased food and water security and subsequent malnutrition [10,36]. Maternal physical risks included gestational hypertension [15,18], alongside broader pregnancy and labor complications—such as gestational diabetes, kidney disease, disruptions in healthcare delivery, cesarean sections, and intrapartum infections [18,28].

Substantial maternal psychological impacts were also documented, including post-traumatic stress, eco-anxiety, climate despair, and heightened climate anxiety [9,17,18,24,27], with Feduniw et al. [24] observing a STAI score change of 1.82 points (95% CI = 0.47–3.18). Regarding perinatal outcomes, studies observed pregnancy loss (miscarriages, abortions, and fetal death) [15,18,23,28] alongside impaired fetal growth [27]. Specific perinatal complications reported by different reviews [14,15,27,28] included increased odds of preterm birth (OR = 1.18; 95% CI = 0.94–1.47) [14], low birth weight (OR = 1.19; 95% CI = 0.83–1.71) [14], and low birth weight for gestational age (OR = 1.25; 95% CI = 1.08–1.43) [14].

#### 3.4.4. Vector-Borne Diseases

Six reviews examined the impact of vector-borne diseases on pregnant women’s health [18,22,23,35,36,39]. Maternal physical complications included gestational hypertension [23] and a strong association with maternal mortality [18]. The expanding geographical distribution of vector-borne pathogens—including Malaria, Dengue, Zika, West Nile virus, and Yellow fever, along with their mosquito and tick vectors—was linked to pregnancy loss (miscarriages and fetal death) as well as congenital neurological disorders depending on the specific infection [22,35]. Furthermore, exposure to these vector-transmitted pathogens was associated with elevated rates of adverse perinatal outcomes, notably low birth weight and preterm birth [22,35,36].

#### 3.4.5. Chemicals

Two reviews [25,37] summarize the impact of chemicals in pregnant women. It was shown that persistent organic pollutants, pesticides and non-persistent chemicals were associated with an elevated risk of preeclampsia [37]. Also, the exposure to black carbon was linked to a higher risk of pregnancy loss (miscarriage and stillbirth) [25]. On the other hand, heavy metals and black carbon were found to be associated with an increased occurrence of gestational diabetes [25,37].

### 3.5. Frequency Mapping of Reported Outcomes

This section presents the maternal and perinatal health outcomes linked to each climate change dimension across the included reviews. Because climate drivers frequently overlap, individual reviews often reported multiple interrelated outcomes. Table 4 shows the review-level frequencies rather than independent estimates of effect. Overall, the dominant health associations varied across dimensions: Air pollution was most frequently linked to gestational hypertensive disorders and developmental deficits in physical, cognitive, and behavioral domains [10,20,21,33]. Extreme heat was predominantly associated with preterm birth, reported across eight reviews [16,19,23,29,30,31,32,36], while natural disasters were most strongly connected to maternal mental health disorders [9,17,18,24,27]. Vector-borne diseases were primarily linked to perinatal complications such as low birth weight and preterm delivery [22,35,36]. Finally, chemical exposures were notably associated with gestational diabetes [25,37]. A comprehensive cross-tabulation of climate dimensions and their corresponding maternal-perinatal outcomes is provided in Table 5.

## 4. Discussion

This scoping review of reviews aimed to synthesize existing knowledge on how the different dimensions of climate change are associated with health outcomes in pregnant women. After reviewing twenty-eight reviews, we found that the climate change dimensions that primarily affect pregnant women are mainly air pollution, extreme heat, natural disasters, vector-borne diseases and chemicals. It is important to remark that vector-borne diseases and chemicals are indirectly driven by climate change, while the other exposures are directly driven by climate change, but in any case, are climate-driven exposures.

The main maternal and perinatal outcomes reported in relation to these climate change exposures are preterm birth and low birth weight. In terms of physical, cognitive and behavioral development issues; hypertensive disorders during pregnancy, gestational diabetes and problems in maternal mental health were frequently reported.

Regarding air pollution, our review showed low birth weight, preterm birth, fetal death, postpartum depression, gestational diabetes, physical, cognitive/behavioral development problems and hypertensive disorders associated principally to PM_2.5_, and also to NO_2_, NO, SO_2_ and CO. Furthermore, it has been reported that, over the course of a year, more than 3% of all preterm births in the United States have been linked to exposure to PM_2.5_, which equates to nearly 16,000 preterm births. The estimated impact of this effect is $5 billion in healthcare costs [40].

The extreme heat was associated with food security problems, dehydration, heat exhaustion and heatstroke, congestive heart failure, congenital anomalies, vitamin D synthesis problems, placental abruption, maternal mental health issues, low birth weight, neonatal stress, cardiovascular episodes, miscarriage, gestational diabetes, hypertensive disorder, fetal death and preterm birth. In 2009, Deschênes et al. [41] examined the impact of climate change on birth weight using data from 37.1 million births, finding that exposure to extremely high temperatures during pregnancy was correlated with lower birth weight, in line with our review. In addition, hypertension, gestational diabetes, and preterm birth, were the most common obstetric outcomes in the different studies included in our review. In addition, the prevalence of heat-related miscarriages is estimated at 3%, with observational data indicating that the associated risk is 26% higher when exposure to extreme heat occurs up to one month before conception [4]. This emphasizes the importance of prevention and early intervention.

Related to natural disasters, the principal maternal and perinatal outcomes observed were reduced fetal growth, water/food security, gestational hypertension, pregnancy complications, labor complications, pregnancy loss and lastly but most recurrently, maternal mental health issues. In contrast, the attention to maternal environmental needs varies across different countries and cultures, as reflected in the action plans developed and political discourses [42]. Perceptions of risk are also shaped by culture [43]. Regrettably, the most vulnerable and underserved populations bear the heaviest toll, particularly in low-income settings, which further widens the socioeconomic gap and inequality.

Vector-borne diseases exacerbated by climate change were associated with gestational hypertension, maternal mortality, pregnancy loss, postpartum infection and perinatal complications. In this line, a study found [44], that dengue infection during pregnancy was associated with elevated risks of miscarriage, preterm birth and low birth weight.

Pregnant women may be exposed to a variety of chemicals present in the environment primarily due to the indirect effects of climate change, such as pesticides, cleaning products, heavy metals (lead and mercury), and VOCs. Exposure to these toxic agents may present risks for pregnancy, such as hormonal disruptions [45]. Additionally, the study by Lamichhane et al. [46] found that higher exposure to phthalates (a group of chemicals primarily used as plasticizers to make plastics more flexible and durable) found in plastics was associated not only with reduced sleep efficiency and duration, but also with prolonged sleep latency during pregnancy.

Despite a growing body of synthesized literature, significant structural and thematic evidence gaps remain across four primary domains. The first is geographical disparities since research remains heavily skewed toward high-income countries in North America and Europe, creating a stark paradox: the regions bearing the highest burden of climate vulnerability—particularly Sub-Saharan Africa, South/Southeast Asia, and Oceania—are the least represented in review-level evidence. The second domain is the understudied climate exposures. While ambient heat and urban particulate matter (PM_2.5_) dominate the literature, slow-onset and compound hazards are critically under-researched. Limited synthesized evidence exists for the maternal-perinatal health impacts of prolonged drought, coastal salinization/saltwater intrusion into drinking water, wildfire smoke plumes, vector ecology shifts, and cascading multi-hazard events. The third domain is maternal and perinatal health outcomes. Synthesized evidence focuses predominantly on acute perinatal endpoints (preterm birth, low birth weight, and stillbirth). Severe maternal morbidity, hypertensive disorders of pregnancy (preeclampsia/eclampsia), maternal mental health (postpartum depression and climate anxiety), and long-term maternal cardiovascular sequelae remain understudied. The last domain is methodological and analytical limitations. The evidence base relies heavily on retrospective cross-sectional and ecological designs, with a notable absence of prospective longitudinal cohorts tracking exposure from preconception through postpartum. Exposure assessment across primary studies relies frequently on coarse gridded climate data or fixed monitoring stations rather than personal, microclimate, or indoor exposure monitoring. There is a near-total absence of synthesized evidence evaluating the efficacy of climate adaptation strategies, early warning systems, or clinical interventions aimed at mitigating climate-related risks in pregnant women.

### 4.1. Strengths and Limitations

The primary strength of this scoping review of reviews lies in its capacity to consolidate a fragmented body of literature into a cohesive, multi-dimensional framework. By synthesizing evidence across disparate environmental drivers—ranging from the physiological impact of extreme heat and air pollution to the psychological repercussions of natural disasters—we provide a holistic map of the obstetric risks inherent to the climate crisis. Including a diverse range of review types—such as narrative, scoping, integrative, systematic, meta-analytic, and umbrella reviews—enabled a holistic mapping of both empirical and conceptual literature. However, this methodological heterogeneity carries distinct implications. Quantitative reviews (e.g., systematic reviews and meta-analyses) provide evaluated evidence on outcomes, whereas narrative and expert reviews offer broader context and conceptual frameworks but often lack systematic appraisal and carry a higher potential for bias. Consequently, synthesized findings were grouped and interpreted with consideration of the underlying methodology of the included reviews, and findings from non-systematic reviews were used primarily for conceptual mapping rather than inferring comparative effectiveness. The diversity in study design, exposure metrics, and reported outcomes reflects the complex, non-linear nature of climate-related health threats. While a quantitative pooling of data was not feasible, this qualitative synthesis offers a superior ‘bird’s-eye view’ of the field, capturing a broader spectrum of maternal-fetal vulnerabilities that a narrower, more homogenous meta-analysis might overlook. Consequently, this approach prioritizes interpretive depth over statistical uniformity, identifying critical gaps in how healthcare professionals must perceive and manage climate-driven risks in clinical practice. Finally, synthesizing evidence across disparate review types (including tertiary evidence from umbrella reviews alongside narrative syntheses) creates potential overlap in primary studies across the included reviews. Readers should interpret the breadth of findings with awareness of the differing methodological standards and potential risk of bias inherent to narrative and non-systematic review formats.

A quantitative overlap metric (such as Pieper’s Corrected Covered Area [CCA]) was not feasible for our dataset, due to the fact that our synthesis includes non-systematic, narrative, and broad scoping reviews that do not provide exhaustive primary study citation lists or standardized citation matrices required for CCA calculations.

Furthermore, considerable variation existed across the included reviews regarding the definitions and operationalizations of both environmental exposures and maternal-perinatal health outcomes. Similarly, health outcomes varied in diagnostic criteria and gestational cutoff points. This inconsistency precludes direct quantitative comparability across reviews.

A methodological limitation of this review relates to the search strategy, which did not include explicit search terms for specific exposures such as agrochemicals, wildfire smoke, droughts, floods, or water insecurity. However, the general terms used in the search strings effectively cover the evidence regarding their impacts on pregnant women. Therefore, the concept of air pollution encompasses both wildfire smoke and pesticide volatilisation. Furthermore, water insecurity, floods, and droughts constitute direct manifestations of climate variability and natural disasters, whose effects on maternal health—such as malnutrition, water contamination by pathogens or chemicals, and physiological overload—are conceptually subsumed under these larger areas, similar to the study of global warming and extreme heat. Consequently, the conceptual framework of the study does not overlook the physiological damage caused by these variables but rather analyzes it in terms of its widest determinants and manifestations, ensuring comprehensive coverage of the state of the art without undermining the rigour of the review. In addition, descriptive mapping indicates that outcome frequencies reflect the number of reviews addressing a specific outcome; however, a higher count of reviews reporting a specific outcome does not necessarily indicate a larger total body of primary evidence or a larger effect size because reviews frequently include overlapping primary studies. Readers should interpret these outcome frequencies as an index of literature focus rather than a weighted quantitative meta-analysis.

Although no language restrictions were applied during database searches, indexing biases in major bibliographic databases predominantly favor English-language journals. This creates potential language and geographic publication biases, which may have led to the omission of relevant regional or non-English syntheses from highly vulnerable Low- and Middle-Income Countries. Furthermore, the synthesis is subject to the publication bias inherent in the primary literature, where studies reporting statistically significant positive associations are more frequently published than those with null or non-significant results.

A minor limitation of this review is that 13 secondary reports could not be retrieved in full text during the screening phase. However, a post hoc examination of the available titles and abstracts for these unretrieved records indicated that their focus (e.g., general thermal stress, extreme weather impacts) aligned closely with topics thoroughly covered by the included full-text reviews. Consequently, it is unlikely that their omission introduced significant reporting bias or altered the core thematic conclusions regarding climate hazards and pregnant women’s health.

Finally, as a scoping synthesis of predominantly observational literature, this review identifies reported statistical associations and risk patterns rather than causal relationships. Confounding variables, such as socio-economic status, baseline maternal health, indoor adaptive capacity, and localized structural inequities, were inconsistently controlled for across the underlying reviews.

### 4.2. Proposed Applications

It is urgent to take specific measures to protect health at this stage of life and to ensure the continuity of health services for the most vulnerable when climate change is linked to health problems. Currently, few climate adaptation measures are tailored to the specific needs of pregnant women, despite their status as a vulnerable population. This must be adequately considered by climate justice [47,48]. To achieve climate justice within healthcare systems, social models of care should be used, focusing on the interdependence of daily life and health, environmental impacts, and health inequalities implementing climate-just care and health promotion key mitigation strategies [49].

Building upon the synthesized evidence of this review, clinical and nursing practices must directly reflect the specific environmental risks documented across the literature. Clinicians—particularly midwives and prenatal nurses—serve as trusted intermediaries positioned to translate this evidence into tailored clinical actions [47,49]. Given the strong evidence linking ambient air pollutants (PM_2.5_, NO_2_) and environmental toxins to gestational diabetes, hypertensive disorders, and neurodevelopmental deficits [9,10,21,33,34], environmental health counselling must be operationalized within standard prenatal visits using targeted intake screenings for high-risk particulate and chemical exposures [3,37]. This directly addresses evidence showing that pregnant women report high educational needs regarding particulate matter and toxic exposures [8]. Furthermore, grounded in synthesized evidence linking extreme heat exposure to higher risks of preterm birth, miscarriage, and maternal cardiovascular stress [4,16,19,31,32,41], healthcare providers must implement proactive heat risk communication. Nursing protocols should equip patients with personalized heat-action plans and hydration strategies well ahead of extreme thermal events [16,40].

In response to evidence linking natural disasters with maternal mental health disruption, labour complications, and reduced fetal growth—which disproportionately impact low-resource settings [5,15,17,27,28,43], healthcare systems must safeguard care continuity. This requires establishing rapid-response protocols, emergency telehealth services, and prioritized outreach for socially vulnerable populations to mitigate disaster-induced adversity [48]. Moreover, aligned with evidence on disaster-related psychological trauma [6,17,24] and findings showing that environmental risk education effectively reduces anxiety in pregnant women [7,8], routine screening for perinatal mental health disorders and eco-anxiety should be integrated into prenatal care alongside formal referral pathways [6]. Thus, drawing on findings that narrative, participatory educational strategies outperform traditional didactic methods in engaging pregnant women [8,47], nursing and prenatal education programs should incorporate interactive modules on air quality, endocrine disruptors, and environmental safety [8,37]. Training midwives and nurses to confidently address these environmental determinants empowers pregnant women, leading to enhanced self-efficacy and the adoption of protective maternal behaviours [8,49].

## 5. Conclusions

Across the included reviews, climate-related exposures were frequently reported in association with adverse maternal and perinatal outcomes. These included reported links between air pollution or chemical exposures and conditions such as hypertension, developmental issues, or gestational diabetes, as well as reported associations between extreme heat or vector-borne diseases and outcomes like preterm birth and low birth weight. Natural disasters were also frequently noted in relation to maternal mental health challenges. Nevertheless, these findings must be interpreted with caution, since the evidence is subject to significant limitations, particularly its reliance on observational designs. Consequently, these results demonstrate observed patterns rather than definitive causal relationships. Despite these limitations, the broad applicability of these findings emphasizes the growing need to integrate climate resilience into clinical and public health practice globally. To apply these findings into clinical care, prenatal health programs—led by midwives and nurses—should incorporate routine environmental risk screenings for toxic exposures, for perinatal eco-anxiety and personalized heat-health action plans. Healthcare systems must also establish disaster-response protocols to protect the most vulnerable pregnant women and ensure continuous climate-just care.

## Figures and Tables

**Table 1:** Search strategy in the different databases.

Database	Search Strategy	Filters
PubMed	(Environmental health[mh] OR Environmental health[tiab]) AND (Pregnant women[mh] OR Maternal health[mh] OR Pregnan*[tiab] OR Maternal health[tiab]) AND (Climate change[mh] OR Global warming[mh] OR Environmental pollution[mh] OR Air pollution[mh] OR Extreme heat[mh] OR Natural disasters[mh] OR Vector borne diseases[mh] OR Climate change[tiab] OR Global warming[tiab] OR Environmental pollution[tiab] OR Air pollution[tiab] OR Extreme heat[tiab] OR Heat wave*[tiab] OR Natural disaster*[tiab])	Review, Scoping Review, Systematic Review
SCOPUS	(INDEXTERMS(Pregnant women) OR INDEXTERMS(Maternal health)) AND (INDEXTERMS(Environmental health) OR TITLE-ABS-KEY(Environmental health) OR INDEXTERMS(Climate change) OR TITLE-ABS-KEY(Climate change) OR INDEXTERMS(Global warming) OR INDEXTERMS(Environmental pollution) OR INDEXTERMS(Air pollution) OR INDEXTERMS(Extreme heat) OR INDEXTERMS(Natural disasters) OR INDEXTERMS(Vector borne diseases) OR TITLE-ABS-KEY(Global warming) OR TITLE-ABS-KEY(Environmental pollution) OR TITLE-ABS-KEY(air pollution) OR TITLE-ABS-KEY(extreme heat) OR TITLE-ABS-KEY(heat wave*) OR TITLE-ABS-KEY(natural disaster*) OR TITLE-ABS-KEY(vector borne disease*))	Review
PsycInfo	(“pregnant women” OR “maternal health”) AND (“environmental health” OR “climate change” OR “Global warming” OR “environmental pollution” OR “air pollution” OR “extreme heat” OR “natural disasters” OR “vector-borne diseases” OR “heat wave*”)	Literature review, Systematic review, Meta-analysis
CINAHL	(MH Expectant Mothers OR AB Maternal health OR AB pregnant wom*) AND (MH Environmental health OR MH Climate change OR MH Greenhouse Effect OR MH Environmental pollution OR MH Air pollution OR MH Extreme Weather OR MH Natural disasters OR MH Vector borne diseases OR AB Climate change OR AB Global warming OR AB Environmental pollution OR AB Air pollution OR AB Extreme heat OR AB Heat wave* OR AB Natural disaster* OR AB Vector borne disease*)	Review, Systematic review
WOS	Title (Expectant Mother* OR Maternal health OR pregnant wom*) AND (Environmental health OR Climate change OR Greenhouse Effect OR Environmental pollution OR Air pollution OR Extreme Weather OR Natural disaster* OR Vector borne disease* OR Global warming OR Extreme heat)	Review article

**Table 2:** Characteristics of the studies included in the review.

Study	Country	Review Design	Number of Studies Included	Exposure Type	Maternal Outcomes	Obstetric Outcomes	Fetal/Neonatal Outcomes
Aktoz et al., 2023 [14]	Turkey	Systematic review + meta-analysis	10	Natural disasters (earthquakes)	Acute stress, compromised health care	Preterm birth	Low birth weight, low birth weight for gestational age
Amekpor et al., 2025 [10]	Ghana	Narrative review	6	Extreme heat	Food security, water security, mortality due to pregnancy, childbirth, postpartum complications		
	Nigeria			Natural disasters			
	USA			Air pollution		Preterm birth	Low birth weight
Asl et al., 2024 [15]	Iran	Systematic review	90	Natural disasters	Gestational hypertension	Preterm birth, miscarriage	Low birth weight, low birth weight for gestational age, fetal death
Baharav et al., 2023 [16]	USA	Review of the current state of science	Not specified	Extreme heat	Gestational hypertension and preeclampsia, gestational diabetes, cardiovascular episodes	Placental abruption, preterm birth, miscarriage	Fetal death
Barkin et al., 2024 [17]	USA	Narrative review	Not specified	Natural disasters	Maternal mental health (eco-anxiety, eco-despair, posttraumatic stress disorder (PTSD), depression and anxiety)		
Braun et al., 2025 [18]	USA	Systematic review	16	Natural disasters	Gestational hypertension, psychological stress, gestational diabetes, kidney disease, infections during labor, and discontinuity of health care.	Cesarean sections, infection during labor preterm birth, abortions	Fetal distress, fetal death, neonatal respiratory disorders, neonatal sepsis, reduced birth weight
				Extreme heat	Gestational hypertension and preeclampsia, gestational diabetes, kidney damage, maternal mental health	Preterm rupture of membranes, uterine bleeding, preterm birth, miscarriages	Low birthweight, meconium aspiration, neonatal jaundice, neonatal intensive care unit admission, congenital defects/conditions (spina bifida)
				Air pollution	Gestational diabetes and hypertension, preeclampsia, mental health complications	Preterm birth	Stillbirth, low birth weight, cardiac, orofacial or limb defects
				Vector-borne diseases	Maternal mortality	Preterm birth, abortion	Congenital Zika syndrome, stillbirth, low birthweight
Carolan-Olah et al., 2014 [19]	USA	Systematic review	8	Extreme heat		Preterm birth	
Chae et al., 2021 [20]	Korea	Systematic review	14	Air pollution	Gestational hypertension		Low birthweight, Impaired lung function, atopic dermatitis, rhinitis, asthma, allergy, respiratory tract infections, low cognition and behavioral problems
Conway et al., 2024 [9]	Switzerland	Umbrella review	79	Extreme heat	Preeclampsia, gestational diabetes, mental health, access to health services	Preterm birth, miscarriage	Stillbirth, congenital anomalies, low birth weight, small-for-gestational age, hospitalization, morbidity, mortality, sudden infant death syndrome
	UK			Air pollution	Hypertension, gestational diabetes, mental health, access to health services	Preterm birth, miscarriage	Stillbirth, intrauterine growth restriction, congenital anomalies, preterm birth, low birth weight, small-for-gestational age, hospitalization, morbidity, mortality
				Natural disasters	Maternal mental health, mortality	Miscarriage, preterm birth	Low birth weight, mortality, morbidity later in life
Decrue et al., 2023 [21]	UK	Narrative review	19	Air pollution (small particles, nitrogen oxides)	Gestational hypertension/preeclampsia, diabetes mellitus, cardiovascular problems during childbirth		
Diouf et al., 2017 [22]	USA	Narrative review	Not specified	Vector-borne diseases		Miscarriage	Intrauterine fetal death, intrauterine transmission of infection, congenital anomalies
Dumbuya et al., 2024 [23]	Algeria	Narrative review	37	Extreme heat		Preterm birth	
				Natural disasters			Fetal death
				Vector-borne diseases	Gestational hypertension		
Feduniw et al., 2022 [24]	Poland	Systematic review + meta-analysis	3	Natural disasters	Anxiety		
Goriainova et al., 2022 [25]	USA	Contemporary Review	31	Chemical products (black carbon)	Gestational diabetes, preeclampsia, increased hair cortisol concentration	Miscarriage, preterm birth, preterm rupture of membranes	Low birth weight, small for gestational age birth, stillbirth, reduced visual motor skills, increase in newborn systolic and diastolic blood pressure
Grabowski et al., 2024 [26]	Poland Switzerland USA Ukraine	Systematic review	69	Air pollution			Small for gestational age, low birth weight
Harville et al., 2010 [27]	USA	Systematic review	49	Natural disasters	Stress	Preterm birth	Reduced fetal growth, low birth weight
Jeffers et al., 2020 [28]	USA	Integrative review	19	Natural disasters	Complications in pregnancy	Preterm birth, cesarean section	Low birth weight, neonatal anomalies, and fetal mortality
Kloog et al., 2019 [29]	Israel	Narrative review	Not specified	Air pollution		Preterm birth	
				Extreme temperatures		Preterm birth	
Kuehn et al., 2017 [30]	USA	Systematic review	28	Extreme heat	Changes in gestational age		Low birth weight, fetal death, and neonatal stress
Lakhoo et al., 2025 [31]	South Africa Australia Belgium UK	Systematic review and meta-analysis	198	Extreme heat	Gestational diabetes, pre-eclampsia and gestational hypertension, emotional stress	Preterm birth, obstetric complications such as placental abruption	Low birth weight, fetal death Congenital anomalies
Marudo et al., 2025 [32]	USA	Expert review	5	Extreme heat	Dehydration, heat exhaustion, heatstroke, gestational hypertension	Preterm birth	Fetal death
Mazumder et al., 2024 [33]	USA Bangladesh	Umbrella review	20	Air pollution	Gestational diabetes, gestational hypertension, pre-eclampsia, gestational hypertension, esophageal atresia and postnatal depression.		
Noorzadeh et al., 2021 [34]	Iran	Narrative review	Not specified	Air pollution	Gestational diabetes, hypertensive disorders of pregnancy	Preterm birth, placental abruption	Congenital malformations, sudden infant death syndrome, stillbirth, low birth weight and head circumference and fetal growth restriction, impaired lung function
Oberlin et al., 2023 [35]	USA	Narrative review	31	Vector-borne diseases	Anemia, preeclampsia	Miscarriage, preterm birth, placental infection	Fetal death, low birth weight, stillbirth, microcephaly and adverse neurologic outcomes, respiratory distress syndrome, hepatosplenomegaly, myocarditis, and meningoencephalitis
Pandipati et al., 2023 [36]	USA	Overview	Not specified	Extreme heat	Congestive heart failure, gestational diabetes, hypertensive disorders, psychotic and neurotic outcomes, homicides and suicides, diminished cognitive ability	Preterm birth, placental abruption, oligohydramnios, uterine contractions	Low fetal weight, fetal death, fetal stress, congenital heart defects
				Air pollution	decreased fertility	Preterm birth	Fetal death, lower respiratory infections, decreased lung function, asthma, atopy, cardiovascular diseases, decreased birth weight
				Natural disasters	Malnutrition, stress, post-traumatic stress disorder, depression, anxiety		
				Vector-borne diseases		Preterm births	Low birth weight, microcephaly, preterm birth
Varshavsky et al., 2020 [37]	USA	Narrative review	64	Chemicals	Preeclampsia, gestational diabetes, and breast cancer		
Vergara-Maldonado et al., 2023 [38]	Chile	Scope review	35	Extreme temperatures	Lower vitamin D Synthesis		
Wiemers et al., 2025 [39]	Germany	Narrative review	Not specified	Vector-borne diseases	Risk of postpartum infection, pre-eclampsia, higher mortality	Miscarriages, preterm birth	Fetal death, low birth weight, intrauterine growth restriction, birth defects, microcephaly, fetal anemia

**Table 3:** Evaluation of the quality of the included studies using the CASPe tool. 1. Was the review conducted on a clearly defined topic? 2. Did the authors search for the appropriate type of articles? 3. Do you think important and relevant studies were included? 4. Do you think the review authors made sufficient effort to assess the quality of the included studies? 5. If the results of the different studies have been combined to obtain a “pooled” result, was it reasonable to do so? 6. What is the overall result of the review? 7. How precise are the results? 8. Can the results be applied in your setting? 9. Were all the important results considered in making the decision? 10. Do the benefits outweigh the harms and costs? Answers: yes/no.

Study	1	2	3	4	5	6	7	8	9	10	Quality
Aktoz et al., 2023 [14]	Yes	Yes	Yes	Yes	Yes	Yes	Yes	Yes	Yes	Yes	High (10/10)
Amekpor et al., 2025 [10]	Yes	Yes	Yes	No	Yes	Yes	No	Yes	Yes	Yes	Moderate (8/10)
Asl et al., 2024 [15]	Yes	Yes	Yes	Yes	Yes	Yes	No	Yes	Yes	Yes	Moderate-High (9/10)
Baharav et al., 2023 [16]	Yes	Yes	Yes	No	Yes	Yes	No	Yes	Yes	Yes	Moderate (8/10)
Barkin et al., 2024 [17]	Yes	Yes	Yes	No	Yes	Yes	No	Yes	Yes	Yes	Moderate (8/10)
Braun et al., 2025 [18]	Yes	Yes	Yes	Yes	Yes	Yes	No	Yes	Yes	Yes	Moderate-High (9/10)
Carolan-Olah et al., 2014 [19]	Yes	Yes	Yes	Yes	Yes	Yes	No	Yes	Yes	Yes	Moderate-High (9/10)
Chae et al., 2021 [20]	Yes	Yes	Yes	Yes	Yes	Yes	No	Yes	Yes	Yes	Moderate-High (9/10)
Conway et al., 2024 [9]	Yes	Yes	Yes	Yes	Yes	Yes	No	Yes	Yes	Yes	Moderate-High (9/10)
Decrue et al., 2023 [21]	Yes	Yes	Yes	No	Yes	Yes	No	Yes	Yes	Yes	Moderate (8/10)
Diouf et al., 2017 [22]	Yes	Yes	Yes	No	Yes	Yes	No	Yes	Yes	Yes	Moderate (8/10)
Dumbuya et al., 2024 [23]	Yes	Yes	Yes	No	Yes	Yes	No	Yes	Yes	Yes	Moderate (8/10)
Feduniw et al., 2022 [24]	Yes	Yes	Yes	Yes	Yes	Yes	Yes	Yes	Yes	Yes	High (10/10)
Goriainova et al., 2022 [25]	Yes	Yes	Yes	No	Yes	Yes	No	Yes	Yes	Yes	Moderate (8/10)
Grabowski et al., 2024 [26]	Yes	Yes	Yes	Yes	Yes	Yes	No	Yes	Yes	Yes	Moderate-High (9/10)
Harville et al., 2010 [27]	Yes	Yes	Yes	Yes	Yes	Yes	No	Yes	Yes	Yes	Moderate-High (9/10)
Jeffers et al., 2020 [28]	Yes	Yes	Yes	No	Yes	Yes	No	Yes	Yes	Yes	Moderate (8/10)
Kloog et al., 2019 [29]	Yes	Yes	Yes	No	Yes	Yes	No	Yes	Yes	Yes	Moderate (8/10)
Kuehn et al., 2017 [30]	Yes	Yes	Yes	Yes	Yes	Yes	No	Yes	Yes	Yes	Moderate-High (9/10)
Lakhoo et al., 2025 [31]	Yes	Yes	Yes	Yes	Yes	Yes	Yes	Yes	Yes	Yes	High (10/10)
Marudo et al., 2025 [32]	Yes	Yes	Yes	No	Yes	Yes	No	Yes	Yes	Yes	Moderate (8/10)
Mazumder et al., 2024 [33]	Yes	Yes	Yes	Yes	Yes	Yes	No	Yes	Yes	Yes	Moderate-High (9/10)
Noorzadeh et al., 2021 [34]	Yes	Yes	Yes	No	Yes	Yes	No	Yes	Yes	Yes	Moderate (8/10)
Oberlin et al., 2023 [35]	Yes	Yes	Yes	No	Yes	Yes	No	Yes	Yes	Yes	Moderate (8/10)
Pandipati et al., 2023 [36]	Yes	Yes	Yes	No	Yes	Yes	No	Yes	Yes	Yes	Moderate (8/10)
Varshavsky et al., 2020 [37]	Yes	Yes	Yes	No	Yes	Yes	No	Yes	Yes	Yes	Moderate (8/10)
Vergara-Maldonado et al., 2023 [38]	Yes	Yes	Yes	No	Yes	Yes	No	Yes	Yes	Yes	Moderate (8/10)
Wiemers et al., 2025 [39]	Yes	Yes	Yes	No	Yes	Yes	No	Yes	Yes	Yes	Moderate (8/10)

**Table 4:** Summary of maternal and perinatal outcomes reported in relation to the different dimensions of climate change.

Dimension of Climate Change	Maternal and Perinatal Outcomes	Number of Reviews That Have Found This Outcome
Air pollution	Postpartum depression	1 [33]
	Low birth weight	1 [26]
	Preterm birth	2 [29,36]
	Fetal death	2 [29,36]
	Gestational diabetes	3 [18,21,33]
	Hypertensive disorders	4 [9,18,21,33]
	Physical, cognitive and behavioral development	4 [10,20,21,34]
Extreme heat	Food security	1 [10]
	Dehydration, heat exhaustion and heatstroke	1 [32]
	Congestive heart failure	1 [36]
	Congenital anomalies	1 [31]
	Vitamin D synthesis	1 [38]
	Placental abruption	1 [16]
	Problems in maternal mental health	1 [18]
	Low birth weight	2 [30,36]
	Neonatal stress	2 [30,36]
	Cardiovascular episodes	2 [16,18]
	Miscarriage	2 [15,17]
	Gestational diabetes	4 [16,18,31,36]
	Hypertensive disorder	4 [16,18,20,32]
	Fetal death	5 [16,30,31,32,36]
	Preterm birth	8 [16,19,23,29,30,31,32,36]
Natural disasters	Reduced fetal growth	1 [27]
	Water and food security	2 [10,36]
	Gestational hypertension	2 [15,18]
	Pregnancy complications	2 [18,28]
	Labor complications	2 [18,28]
	Perinatal complications	4 [14,15,27,28]
	Pregnancy loss	4 [15,18,23,28]
	Problems in maternal mental health	5 [9,17,18,24,27]
Vector-borne diseases	Gestational hypertension	1 [23]
	Maternal mortality	1 [18]
	Pregnancy loss	2 [22,35]
	Postpartum infection	2 [22,35]
	Perinatal complications	3 [22,35,36]
Chemicals	Preeclampsia	1 [37]
	Pregnancy loss	1 [25]
	Gestational diabetes	2 [25,37]

Note: Frequencies are unweighted review-level counts and do not account for review design, methodological quality, overlap of primary studies, effect magnitude, or certainty of evidence.

**Table 5:** Relationship between climate change dimensions and maternal and perinatal outcomes.

	Dimensions of Climate Change				
Maternal and Perinatal Outcomes	Air Pollution	Extreme Heat	Natural Disasters	Vector-Borne Diseases	Chemicals
Pregnancy complications	X	X	X	X	X
Labor complications		X	X	X	
Perinatal complications	X	X	X	X	
Pregnancy loss	X	X	X	X	X
Problems in maternal mental health	X		X		
Food and water security		X	X		

X: It shows that the climate change dimension in the column has been linked to the maternal or perinatal outcome indicated in the selected row.

## Data Availability

The full data presented in the study are openly available in the Zenodo repository at https://doi.org/10.5281/zenodo.20355035.

## Supplementary Materials

The following supporting information can be downloaded at: https://www.mdpi.com/article/10.3390/nursrep16090332/s1, File S1: Preferred Reporting Items for Systematic Reviews and Meta-Analyses extension for Scoping Reviews (PRISMA-ScR) Checklist.

## Public Involvement Statement

No public involvement in any aspect of this research.

## Guidelines and Standards Statement

This manuscript was drafted against the Preferred Reporting Items for Systematic reviews and Meta-Analyses extension for Scoping Reviews (PRISMA-ScR) Checklist [10].
